# Effect of an office worksite-based yoga program on heart rate variability: outcomes of a randomized controlled trial

**DOI:** 10.1186/1472-6882-13-82

**Published:** 2013-04-10

**Authors:** Birinder S Cheema, Angelique Houridis, Lisa Busch, Verena Raschke-Cheema, Geoff W Melville, Paul W Marshall, Dennis Chang, Bianca Machliss, Chris Lonsdale, Julia Bowman, Ben Colagiuri

**Affiliations:** 1 School of Science and Health, University of Western Sydney, Penrith, NSW, 2751, Australia; 2 Centre for Complementary Medicine Research, University of Western Sydney, Campbelltown, NSW, 2751, Australia; 3 Yoga Synergy Pty Ltd, Bondi Junction, NSW, 2022, Australia; 4 School of Psychology, University of Sydney, Sydney, NSW, 2006, Australia

**Keywords:** Stress, Quality of life, Anxiety, Physical fitness, Mood

## Abstract

**Background:**

Chronic work-related stress is an independent risk factor for cardiometabolic diseases and associated mortality, particularly when compounded by a sedentary work environment. The purpose of this study was to determine if an office worksite-based *hatha* yoga program could improve physiological stress, evaluated *via* heart rate variability (HRV), and associated health-related outcomes in a cohort of office workers.

**Methods:**

Thirty-seven adults employed in university-based office positions were randomized upon the completion of baseline testing to an experimental or control group. The experimental group completed a 10-week yoga program prescribed three sessions per week during lunch hour (50 min per session). An experienced instructor led the sessions, which emphasized *asanas* (postures) and *vinyasa* (exercises). The primary outcome was the high frequency (HF) power component of HRV. Secondary outcomes included additional HRV parameters, musculoskeletal fitness (i.e. push-up, side-bridge, and sit & reach tests) and psychological indices (i.e. state and trait anxiety, quality of life and job satisfaction).

**Results:**

All measures of HRV failed to change in the experimental group versus the control group, except that the experimental group significantly increased LF:HF (p = 0.04) and reduced pNN50 (p = 0.04) versus control, contrary to our hypotheses. Flexibility, evaluated *via* sit & reach test increased in the experimental group versus the control group (p < 0.001). No other adaptations were noted. *Post hoc* analysis comparing participants who completed ≥70% of yoga sessions (n = 11) to control (n = 19) yielded the same findings, except that the high adherers also reduced state anxiety (p = 0.02) and RMSSD (p = 0.05), and tended to improve the push-up test (p = 0.07) versus control.

**Conclusions:**

A 10-week *hatha* yoga intervention delivered at the office worksite during lunch hour did not improve HF power or other HRV parameters. However, improvements in flexibility, state anxiety and musculoskeletal fitness were noted with high adherence. Future investigations should incorporate strategies to promote adherence, involve more frequent and longer durations of yoga training, and enrol cohorts who suffer from higher levels of work-related stress.

**Trial registration:**

ACTRN12611000536965

## Background

Epidemiological investigations have consistently shown that chronic work-related stress can increase the risk of cardiometabolic diseases by as much as 50% [1,2]. This link between stress and disease is influenced by autonomic imbalance [3] implicating an overactive or dysregulated sympathetic nervous system (SNS) and hypothalamus-pituitary-adrenal (HPA) axis [4,5].

Cortisol, the main effector of SNS and HPA axis activation, functions to physiologically prepare the body for physical exertion (i.e. “fight or flight”) when a threat is perceived. However, the absence of physical exertion elicits acute hyperlipidemia and hyperglycemia which, over time, can contribute to the genesis of more advanced chronic diseases, including coronary artery disease and type 2 diabetes [6]. Chronic work-related stress is therefore particularly concerning in individuals employed in low physical activity occupations, such as in office workers.

*Hatha* yoga is an ancient physical practice that emphasizes the performance of exercises (*vinyasa*) and postures (*asanas*) [7]. Participation in *hatha* yoga has increased markedly in the West in recent decades [8] and empirical investigations have shown that several weeks to months of training can improve markers of physical fitness [9], musculoskeletal pain [10], psychological stress (i.e. depression, anxiety) [11], and health-related quality of life (QoL) [12]. Hence, many of the benefits of *hatha* yoga training are particularly relevant to sedentary office workers.

Heart rate variability (HRV) is the instantaneous variation in heart rhythm due to autonomic influences on the sinoatrial node. Low HRV indicates high sympathetic- and low parasympathetic (vagal) autonomic activity, and is an established predictor of cardiac events [13] and mortality [14,15]. Notably, excess work-related stress in office workers has been shown to reduce HRV acutely [16]. By contrast, a single session of *hatha* yoga can acutely improve HRV in experienced practitioners [17] and we recently reported a similar effect in non-experienced participants performing 15 min of yoga postures while seated at their desk [18].

Despite this evidence of an acute effect [18] there are currently limited data to suggest that *hatha* yoga training can induce chronic adaptation of HRV [19]. Randomized controlled trials have shown that prolonged aerobic exercise training (e.g. cycling, running) can improve HRV at rest [20] and given that *hatha* yoga is thought to induce benefits similar to those achieved with conventional exercise [21] such investigation is warranted. The improvement of HRV at rest would indicate greater parasympathetic nervous system activity and therefore reduced risk of cardiovascular morbidity and mortality.

It is oftentimes difficult for individuals to engage in therapeutic practices due to time restrictions incurred by work and family life. The integration of yoga practice into the office workplace could provide a practical method of mitigating chronic, work-induced stress and inactivity. Therefore, we hypothesized that a 10-week, worksite-based *hatha* yoga program would improve HRV and that this adaptation would be concomitant with other health-related benefits, including improvements in musculoskeletal fitness, anxiety, job satisfaction and QoL, in a cohort of office workers.

## Methods

### Study design

The methodology has been presented in full elsewhere [22]. Briefly, this trial compared the outcomes of participants randomized to an experimental treatment group (*hatha* yoga) with those assigned to a no-treatment control group. The intervention period was 10 weeks, with primary and secondary outcomes collected prior to and following the intervention period (week 0 and week 11). The University of Western Sydney Human Research Ethics Committee approved all procedures and the trial was registered with the *Australian New Zealand Clinical Trials Registry*. All data were collected at the University of Western Sydney from March to June 2011.

### Randomization

Participants were randomized *via* computer-generated randomly permuted blocks stratified by gender (http://www.randomization.com). An investigator not involved in data collection prepared the assignments in sealed envelopes that were given to participants following the completion of baseline testing.

### Participants

#### Eligibility criteria

Adult (>18 years); employed as a full-time academic staff, general staff or post-graduate student at the University of Western Sydney; not currently engaged in regular yoga practice; available to attend three yoga sessions per week during lunch break; able to communicate in English; no acute or chronic medical conditions that would contraindicate the performance of *hatha* yoga practice [23].

### Interventions

#### Experimental group

Participants in the experimental group engaged in a 10-week *hatha* yoga program delivered at their place of work (University of Western Sydney, Campbelltown Campus). Sessions were group-based, prescribed three sessions per week during lunch hour (50 min per session) and led by an experienced instructor from Yoga Synergy Pty. Ltd. (Sydney, Australia). The yoga program was developed to teach beginner students safely and progressively over the intervention period and was based on the *Yoga Synergy Water Sequence*, created by Simon Borg Olivier and Bianca Machliss. Approximately 95% of each session involved the performance of *asanas* and *vinyasa*. Each session began with an introductory series of movements designed to warm-up the large joints, spine, and extremities (*utkata vinaysa* and *baddha hasta meru danda vinyasa*). This was followed by salute to the moon (*candra namaskar*). Following this, a sequence of standing poses which linked to one another (using *vinyasas*) were done to bring heat to the body, whilst stretching and strengthening the muscles (*Vinyasas*: *Trikonasa*, *Parsvakona*, *Gadja Hasta Padottanasana, Gadja Baddha Padottanasana, Parsvottana, Eka Pada, Virabhadra*). Floor postures followed the standing postures; these were also linked together in *vinyasas* and included: forward bends, twists, hip opening, backarches and releases from backarches (*Pashima, Janu Sirsa, Baddha Kona, Urdhva Dhanura, Jathara, Hasta*). As training progressed, an inversion (shoulderstand) was added to bring calm and restore the body and nervous system. After shoulderstand a series of neck and spine releases were performed to ensure safety. The participants then sat on the floor to engage in a few minutes of breathing exercises (*pranayama*) and supine meditation/relaxation (*savasana*). Most of the postures and exercises involved a simple and more challenging version. Participants were instructed to choose the level of difficulty appropriate to them during any given session. All participants were instructed to wear appropriate clothing for the yoga session, and change facilities were available at the venue.

#### Control group

Participants in the control group were advised to maintain current lifestyle practices and were not provided specific information or instructions about yoga practice.

### Outcome measures

Outcome measures were collected at baseline (week 0) and after the intervention period (week 11) within a single testing session. Qualified and experienced personnel blinded to the group assignment assessed HRV and musculoskeletal fitness. The psychological questionnaires were self-administered.

#### HRV and heart rate

HRV was evaluated in a quiet, temperature-controlled room in accordance with procedures developed by the *Task Force for Pacing and Electrophysiology*[24]. Participants were advised to abstain from caffeinated food and beverages on the day of the assessment and avoid exercise for at least 24 hours prior to the assessment. Repeat assessments (week 11) were completed at precisely the same time of day and using the same procedures as the baseline assessment (week 0) and at least 48 hours following the final yoga session in those randomized to the experimental group. Following 15 minutes of supine rest with a regular and calm breathing pattern, a continuous 10-minute ECG recording was collected using the Sphygmocor system and HRV software (Sphygmocor, AtCor Medical Pty, Sydney, Australia). Participants were instructed not to speak and to use a regular and calm breathing pattern during the entire assessment. The primary outcome of this study was the high frequency (HF) spectral power component of HRV (measured in absolute units; ms^2^) given that parasympathetic nervous system (vagal) activity is the major contributor to the HF component [24]. The following time domain parameters of HRV were calculated from RR intervals: standard deviation of the NN intervals (SDNN), root-mean-square of the successive normal sinus RR interval difference (RMSSD) and the percentage of absolute differences between successive normal RR intervals that exceed 50 ms (pNN50). Frequency domain variables, including total power, HF and low frequency (LF) power (measured in ms^2^) and the LF:HF ratio were derived from spectral analysis of successive R-R intervals. Heart rate was evaluated by computing the average number of beats per minute during the 10 minute recording.

#### Musculoskeletal fitness

Upper-body muscular endurance was evaluated using a standardised push-up test, according to procedures outlined by the *American College of Sports Medicine*[23]. Low-back and abdominal endurance was evaluated by means of an isometric, side-bridge test [25]. Time to exhaustion was computed for both the left and right side and the average score was reported. Low-back and hip flexibility was evaluated *via* standardized sit-and-reach test [23].

#### Psychological health status

The *Medical Outcomes Trust Short-form 36 Health Status Questionnaire (SF36)* Version 1.0, a widely used and validated questionnaire [26] was used to assess eight domains and two summary scores of health-related QoL. The *State-Trait Anxiety Inventory* (STAI), a widely used and validated inventory that consists of two, 20-item self-report scales, was used to assess state anxiety and trait anxiety as distinct and clearly defined psychological constructs in adults [27]. Scores range from 20 to 80 and lower scores are indicative of lower anxiety. Job satisfaction was evaluated *via* the *Job Descriptive Index* (JDI) and the *Job in General* (JIG) scale, both validated for use in the general population, including office workers [28]. The JDI assesses five perceptions of job satisfaction: supervision, co-workers, work, pay and promotion. The JIG assesses general job satisfaction.

### Demographics and health status

Demographic and health status data were extracted during the recruitment screening process and baseline testing by means of standardized questionnaires and assessments. Factors included age, occupation, height, weight, resting blood pressure, waist circumference, smoking history, medical history, and medication usage. Changes in health status, including adverse events, were during the intervention period monitored by means of a structured questionnaire administered weekly *via* email.

### Statistical analyses

Analyses were performed using the Statistical Package for the Social Sciences (IBM©, SPSS Version 19.0). All data were inspected visually and statistically for normality (skewness and kurtosis between -1 and +1). Non-normally distributed data were log-transformed prior to entry into parametric models. Pearson product–moment correlation coefficient tests (*r*) were computed between all HRV indices and heart rate using data for the total cohort at baseline. Correlations (*r*) were also computed between state anxiety and measures of HRV in the total cohort using baseline data to investigate relationships between psychological and physiological stress. Differences between the yoga and control group at baseline were investigated using an independent samples *t*-test and chi square test, for continuous and categorical data, respectively. All participant data were included in the primary (group × time) analyses regardless of compliance to yoga intervention. Missing data were imputed using the last-observation-carried-forward method (intention-to-treat). Changes between the yoga and control group were determined by analysis of covariance (ANCOVA) of the post-treatment score controlling for the baseline score. Additional covariates were identified by comparison of group means at baseline for statistical and/or clinically meaningful differences (i.e. BMI). A p value of <0.05 was considered indicative of statistical significance.

## Results

### Flow of participants

**Figure 1:**
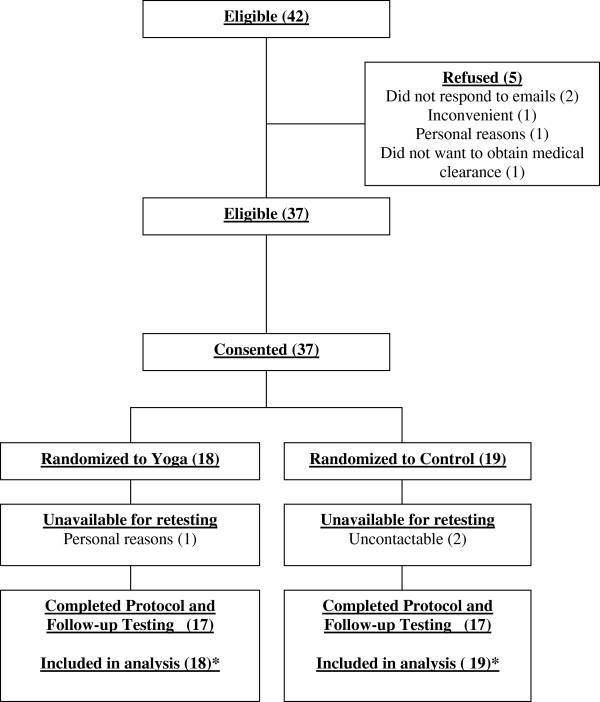
**Flow of participants.** * Baseline data carried forward for 1 yoga and 2 control participants lost to follow-up.

Forty-two individuals (n = 42) expressed an interest in the study, and were deemed eligible; five withdrew prior to baseline testing with reasons presented in Figure 1. Thirty-seven individuals provided written informed consent, completed baseline testing, and were randomized to the yoga (n = 18) or control group (n = 19). One participant in the yoga group withdrew in week 2 for personal reasons, while two in the control group failed to complete follow-up testing. Recruitment resulted in lower participant interest and enrolment than anticipated [22], and therefore our *a priori* calculation of statistical power was reduced from 80% to 63%.

### Baseline characteristics

Participant characteristics are presented in Table 1. No statistically significant differences were observed between groups at baseline on the descriptive characteristics presented. However, due to potentially clinically important differences in BMI (p = 0.09), this variable was included as a covariate in all ANCOVA models. The cohort ranged in age from 21 to 58 years. BMI ranged from 18.3 to 39.2 kg/m^2^, and seven participants fulfilled the clinical criteria for obesity (i.e. BMI ≥30.0 kg/m^2^). Two participants were current smokers, while one had a history of tobacco use but did not currently smoke. Other than obesity, the most common chronic conditions were asthma (n = 4), hypercholesterolemia (n = 4) and hypertension (n = 5). None of the participants were presently engaged in regular yoga practice but nearly half had prior experience at a beginner level (17/37, 46%). The majority of participants were administrative staff, technical support staff, or post-graduate students.

**Table 1:** Baseline characteristics of the total cohort and groups

**Characteristic**	**Total cohort (n = 37)**	**Yoga group (n = 18)**	**Control group (n = 19)**
Age (y)	38 ± 12	37 ± 12	39 ± 13
Women:Men	30:7	14:4	16:3
Body weight (kg)	68.6 ± 11.8	68.1 ± 10.7	69.1 ± 13.0
Height (cm)	165 ± 9	167 ± 9	163 ± 7
Body Mass Index (kg/m^2^)	25.3 ± 4.8	24.4 ± 4.0	26.1 ± 5.6
Systolic blood pressure (mmHg)	121 ± 12	122 ± 14	121 ± 9
Diastolic blood pressure (mmHg)	79 ± 10	79 ± 11	80 ± 8
Occupation (n):			
• Administrative staff	13	7	6
• Technical support	10	4	6
• Post-graduate student	8	3	5
• Academic staff	5	3	2
• Research staff	1	1	0

Data presented as mean ± standard deviation.

### Correlations between measures of HRV at baseline

Correlation coefficients relating to measures of HRV in the total cohort at baseline, after normalization of data, are presented in Table 2. Log SDNN, log RMSSD, pNN50, log LF power, log HF power and log total power were positively, significantly and highly correlated (all p < 0.001; all *r* ≥0.69). Log LF:HF was negatively, significantly and moderately correlated with pNN50 (p = 0.02), log RMSSD (p = 0.01) and log HF power (p < 0.001). Resting heart rate was significantly, negatively and moderately correlated with log SDNN (p = 0.004), log RMSSD (p = 0.001), pNN50 (p = 0.003), log LF power (p = 0.02), log HF power (p = 0.003) and log total power (p = 0.01).

**Table 2:** Correlations among measures of heart rate variability and heart rate

	**Log SDNN**	**Log RMSSD**	**pNN50**	**Log LF power**	**Log HF power**	**Log total power**	**Log LF:HF**	**Resting HR**
Log SDNN		0.91^*^	0.76^*^	0.75^*^	0.76^*^	0.83^*^	−0.23	−0.48^*^
Log RMSSD			0.81^*^	0.69^*^	0.84^*^	0.83^*^	−0.44^*^	−0.57^*^
pNN50				0.73^*^	0.85^*^	0.82^*^	−0.41^*^	−0.48^*^
Log LF Power					0.80^*^	0.92^*^	0.06	−0.39^*^
Log HF Power						0.94^*^	−0.56^*^	−0.49^*^
Log Total Power							−0.28	−0.44^*^
Log LF:HF								0.27

*p < 0.05; *SDNN*, standard deviation of the normal-normal interval; *RMSSD*, root-mean-square of the successive normal sinus RR interval difference; *pNN50*, percentage of absolute differences between successive normal RR intervals that exceed 50 ms; *HF*, high frequency power; *LF*, low frequency power; *LF:HF*, ratio of low frequency to high frequency power; *HR*, heart rate.

### Correlations between state anxiety and HRV measures

Higher levels of state anxiety were associated with higher resting heart rate (r = 0.51, p = 0.002), lower pNN50 (r = -0.36, p = 0.03), lower log RMSSD (r = -0.39, p = 0.02), lower log LF power (r = -0.344, p = 0.05), lower log HF power (r = -0.47, p = 0.01), and lower log total power (r = -0.40, p = 0.02).

### Attendance and adverse events

Attendance in the yoga group ranged from 33% to 97% and averaged 73 ± 19%. Eleven participants (11/17, 65%) attended ≥70% of the sessions. No adverse events were documented in either the experimental or control group during the study.

### Outcome measures

All outcome measures are presented in Table 3.

**Table 3:** Summary of primary and secondary outcome measures

	**Yoga group**		**Control group**		**Effect size**	p^*^
**Outcome measure**	**Week 0**	**Week 11**	**Week 0**	**Week 11**		
***HRV and Heart Rate***						
Log HF Power	2.75 ± 0.63	2.51 ± 0.83	2.57 ± 0.58	2.53 ± 0.55	0.02	0.48
Log SDNN	1.81 ± 0.21	1.76 ± 0.28	1.69 ± 0.19	1.68 ± 0.19	0.01	0.70
Log RMSSD	1.73 ± 0.32	1.59 ± 0.42	1.58 ± 0.29	1.62 ± 0.27	0.05	0.22
pNN50	27.7 ± 27.5	20.7 ± 25.3	16.0 ± 16.6	19.4 ± 18.4	0.12	0.04
Log LF Power	2.89 ± 0.57	2.86 ± 0.73	2.61 ± 0.45	2.55 ± 0.49	0.01	0.53
Log Total Power	3.34 ± 0.50	3.26 ± 0.64	3.18 ± 0.42	3.09 ± 0.42	0.01	0.66
Log LF:HF	0.13 ± 0.33	0.35 ± 0.43	0.04 ± 0.40	0.04 ± 0.38	0.12	0.04
Heart Rate (beats/min)	62 ± 6	65 ± 9	68 ± 10	67 ± 9	0.07	0.13
***Musculoskeletal Fitness***						
Sit and reach (cm)	27.8 ± 10.0	31.0 ± 8.7	27.3 ± 11.0	26.4 ± 10.4	0.35	<0.001
Push-up (reps)	17 ± 10	21 ± 11	17 ± 13	20 ± 13	0.04	0.27
Side bridge (s)	60.1 ± 35.3	60.8 ± 38.7	47.0 ± 27.2	42.9 ± 24.7	0.01	0.59
***Psychological Measures***						
Trait Anxiety (STAI)	36.1 ± 10.4	33.1 ± 9.7	38.6 ± 7.8	35.3 ± 7.2	0	0.96
State Anxiety (STAI)	32.7 ± 6.2	27.9 ± 7.6	36.1 ± 9.7	32.1 ± 7.8	0.04	0.25
General Job Satisfaction (JIG)	46.9 ± 7.6	47.1 ± 7.7	46.3 ± 6.0	46.7 ± 6.2	0.01	0.52
PCS (SF36)	49.4 ± 2.9	47.5 ± 2.7	47.4 ± 5.1	46.9 ± 4.8	0.04	0.27
MCS (SF36)	45.3 ± 8.9	47.5 ± 10.6	45.2 ± 923	48.5 ± 7.7	0.01	0.66

Data presented as mean + standard deviation.

^*^Estimates based upon the final model including the baseline assessment value, age and body mass index as covariates.

*SDNN*, standard deviation of the normal-normal interval; *RMSSD*, root-mean-square of the successive normal sinus RR interval difference; *pNN50*, percentage of absolute differences between successive normal RR intervals that exceed 50 ms; *HF*, high frequency power; *LF*, low frequency power; *LF:HF*, ratio of low frequency to high frequency power; *STAI*, State-trait anxiety inventory; *JIG*, Job in general scale; *SF36*, medical outcomes trust short-form 36 health status questionnaire; *PCS*, physical component scale; *MCS*, mental component scale.

#### HRV and heart rate

Log HF, the primary outcome, did not significantly improve in the yoga group versus the control group over time (p = 0.48). Contrary to our hypotheses, the yoga group significantly reduced pNN50 (p = 0.04) and increased log LF:HF (p = 0.04) versus the control group. No group × time interaction effects were noted for log SDNN, log RMSSD, log LF power, log total power or resting heart rate (Table 3).

#### Musculoskeletal fitness

Low back and hip flexibility, evaluated *via* sit and reach test, significantly increased in the yoga group versus the control group following the intervention period (p < 0.001). No group × time interaction effect was noted for the push-up test or the side-bridge test (Table 3).

#### Psychological health status

None of the QoL domain scores changed significantly in the yoga group versus the control group following the intervention period (data not shown, all p > 0.05), nor were group × time effects noted for the QoL summary scales (i.e. Physical Component Scale and Mental Component Scale; Table 3). Both groups reduced state and trait anxiety measures from pre to post intervention; however, no significant group × time interaction effects were noted. None of the five domains of job satisfaction measured *via JDI* changed significantly between groups over time (data not shown, p > 0.05), nor did general job satisfaction measured *via JIG* (Table 3).

### Post hoc analyses

*Post-hoc* analyses were conducted to evaluate the effect of adherence on adaptation. ANCOVA of the post-treatment score controlling for the baseline score, age and BMI were computed for all outcome measures between participants who attended ≥70% of the yoga sessions (n = 11) versus the control group (n = 17). The findings remained unchanged from the primary analysis, except that the high adherers to yoga significantly reduced state anxiety (p = 0.02) and RMSSD (p = 0.05), and tended to improve the push-up test (p = 0.07) versus the control group.

## Discussion

This study evaluated the effect of an office worksite-based *hatha* yoga program on HRV and related outcomes in a cohort of office workers. The findings suggest that *hatha* yoga prescribed three sessions per week during lunch hour (50 min/session) for 10 weeks is insufficient to improve vagal tone reflected *via* HF power and related HRV measures. However, the yoga intervention did significantly increase low-back and hip flexibility (p < 0.001) and *post hoc* analyses revealed that high adherers to yoga reduced state anxiety (p = 0.02) and tended to improve muscular endurance (p = 0.07).

Studies have shown that a single session of *hatha* yoga can increase HRV acutely [17,18], with such adaptation hypothesized to be mediated by reduced respiration [18]. However, there are currently limited data to suggest that prolonged training can induce adaptation of resting HRV. Telles *et al.*[29] investigated the effect of a *hatha* yoga intervention prescribed daily for seven days in 22 male survivors of the 2008 Bihar flood. Each yoga session was 60 min and involved exercises, postures, breathing and a brief meditation period, similar to our intervention. The yoga group (n = 11) failed to improve any parameter of HRV (i.e. LF and HF power, LF:HF and pNN50) versus the control group (n = 11). This lack of change may have been due to the short intervention period (one week), small sample size, and potentially low statistical power. Bowman *et al.*[19] prescribed 6 weeks of yoga training that involved postures, breathing and relaxation in 12 healthy older adults. The number of sessions per week was not specified. The authors noted a significant improvement in the mid frequency component but not the HF component of HRV following yoga intervention. No other HRV parameters were measured. To our knowledge, no additional trials have investigated the effect of *hatha* yoga training on HRV at rest.

Studies of aerobic exercise training (e.g. cycling and running) have been shown to increase HF power and other measures of HRV at rest [20]. This adaptation may be mediated by the training-induced reduction of resting heart rate, influenced by such factors as cardiac muscle remodeling (i.e. left ventricular volume hypertrophy, and increased end-diastolic volume and stroke volume) [20]. Reductions of resting heart rate appear to be associated with reductions of efferent sympathetic neural outflow to the sinoatrial node, and hence higher HRV [20,30]. For example, bradycardia in elite endurance-trained athletes with high cardiac outputs is associated with high resting HRV [31].

In the present study, resting heart rate at baseline was found to be significantly and inversely proportional to all relevant measures of HRV, as expected (Table 2). However, unexpectedly, the yoga group tended to increase resting heart rate versus the control group (p = 0.13), and hence favourable adaptation of the cardiovascular system was not achieved. Additional anomalous findings included the reduction of pNN50 (p = 0.04) and the increase of LF:HF (p = 0.04) in yoga versus control (Table 3). The *post hoc* analyses of high adherers also revealed that RMSSD was reduced in yoga versus control (p = 0.05), contrary to our hypotheses. These adaptations, which indicate increased sympathetic- and reduced parasympathetic influence on the heart, are difficult to interpret. Regression of resting heart rate to the mean may have contributed to the effects. Notably, resting heart rate was similar between groups post intervention (Table 3).

By contrast to our findings, the yoga intervention prescribed by Bowman *et al.*[19] significantly reduced resting heart rate (p < 0.05). This disparity between trials could be attributed to a many factors, including differences between interventions, measurement techniques, and cohorts. Older adults, such as those enrolled by Bowman *et al.*[19] (aged 62-81 years) typically have lower levels of physically fitness than younger adults such as those enrolled in the present study (aged 21-58 years) and hence, given greater potential for improvement, could respond more rapidly and favourably to the yoga intervention. Future trials enrolling relatively young and healthy adults may require longer, more frequent, and more intense *hatha* yoga training to induce desired improvements in HRV.

Alternatively, randomized controlled trials have shown that HRV can be improved with the non-physical aspects of yoga (i.e. breathing and meditation) [32,33]. Santaella *et al.*[32] prescribed four months of specific breathing exercises (*bhastrika*) in elderly participants (>60 years) and documented a significant decrease in the LF:HF ratio, while Paul-Labrador *et al.*[33] prescribed transcendental meditation twice per week for 16 weeks in patients with coronary artery disease and detected an increase in HF power (p = 0.07). Mechanisms underlying the positive adaptation of HRV parameters in these trials [32,33] were not elucidated. However, the enhancement of LF:HF noted by Santaella *et al.*[32] likely occurred independent of changes in the resting respiration rate, given that breathing was paced at 12 cycles/min (0.2 Hz) during the HRV assessment [32]. Paul-Labrador *et al.*[33] suggested that neurohumoral factors might be responsible for the enhancement of HRV parameters secondary to their meditation intervention but provided no further interpretation. Robust trials are currently required to investigate the therapeutic effectiveness of chronic yogic breathing and meditation practices performed in the office workplace, as well as the physiological mechanisms underlying adaptation. Such trials will have to consider respiration rate as a confounding variable. We did not prescribe paced breathing during HRV assessment and this may be seen as a limitation in terms of mechanistic exploration.

Our primary analysis revealed that low-back and hip flexibility, evaluated *via* the sit and reach test, significantly improved with the worksite-based yoga intervention versus control (Table 3). This is an important finding given that sedentary work can reduce flexibility, and may increase the risk of musculoskeletal injuries and disability. Improvements of flexibility and related musculoskeletal symptoms secondary to *hatha* yoga training have been well documented [10,34].

Additional measures of musculoskeletal fitness (i.e. push-up test and side-bridge test) did not significantly improve in the yoga group as compared to control. However, we did detect a trend toward enhanced upper-body muscular endurance in high adherers (p = 0.07). Studies have shown that *hatha* yoga can significantly improve measures of muscular endurance and strength [9,35]. These adaptations are influenced by the enhancement of muscle size and quality, measures that decline as a consequence of aging and physical inactivity. Trials investigating larger doses of *hatha* yoga in the workplace (e.g. greater frequency, intensity and duration of training) might induce significant adaptations of muscular fitness and physiology, changes that would be highly beneficial to individuals employed in sedentary occupations.

State anxiety measures in the total cohort at baseline were found to be directly proportional to resting heart rate and inversely proportional to pNN50, log RMSSD, log LF power, log HF power, and log total power (all p < 0.05). These relationships support an association between psychological and physiological stress. Both state and trait anxiety failed to improve in the yoga group versus the control group (Table 3). However, *post hoc* analyses showed that higher adherence to the yoga intervention significantly reduced state anxiety (p = 0.02). The reduction of state anxiety represents an improvement in the ability to manage and respond to stressors. This is a key finding given the link between stress and disease [36,37]. Previous research suggests that yoga might be particularly effective for targeting anxiety [12,38] and anxiety-related disorders [39]. Additional robust trials involving enrolling cohorts suffering from anxiety are warranted [39], and it is recommended that such investigations prescribe practical interventions within the workplace, and explore relationships between changes in anxiety and changes in physiological markers of stress. Our data suggest that psychological improvements may precede improvements of HRV.

Domains of QoL and job satisfaction failed to change significantly between groups over time. Investigations have shown that *hatha* yoga can induce significant improvements in many aspects of QoL. However many of these studies involved chronically diseased [40] and elderly cohorts [41,42] who tend to suffer impairments of QoL. Our study cohort possessed higher levels of QoL compared to normative data from the *Australian Bureau of Statistics*[43] and hence may have had limited potential for improvement. Studies that have noted significant improvements in QoL in apparently healthy adults have generally involved longer durations of *hatha* yoga training (e.g. 16 weeks to 6 months) [44,45]. Given that QoL and job satisfaction are related [46], interventions that optimally target QoL may also improve perceptions of job satisfaction.

The strengths of this trial included the study design and the practical nature of the intervention. Yoga can induce significant and broad-ranging health benefits [21] and requires minimal financial investment. Hence, the integration of yoga into the workplace setting as a cost-effective means of promoting health and well being in employees makes intuitive sense. The limitations of this study include the single worksite investigated (university setting) and the low number of participants enrolled. It is possible that this study suffered from low statistical power; however, significant improvements in HRV parameters have been noted in studies of yoga involving fewer participants [19,32].

## Conclusions

In summary, a 10-week *hatha* yoga intervention delivered at the office worksite during lunch hour did not improve HF power or other HRV parameters. However, improvements in flexibility, state anxiety and musculoskeletal fitness were noted with high adherence. Future investigations should incorporate strategies to promote adherence, involve more frequent and longer durations of yoga training, and enrol cohorts who suffer from higher levels of work-related stress. The exact prescriptions of *hatha* yoga required to favourably reduce cardiometabolic disease risk in sedentary workers should be the topic of continued investigation.

## Competing interests

BM is owner of a commercial Yoga studio, Yoga Synergy Pty Ltd. All other authors declare that they have no competing interests.

## Authors’ contributions

BSC conceived and designed the study and drafted the manuscript. AH, LB and GM were involved in the acquisition of all data and drafting of the manuscript. VRC organised the research group, provided consultation regarding the yoga intervention and drafted the manuscript. PWM provided consultation regarding physical functioning outcome measures. DC provided consultation regarding heart rate variability method. BC, CL and JB provided consultation regarding statistics and psychological outcome measures. BM designed the yoga intervention. All authors have read and approved the final manuscript.

## Pre-publication history

The pre-publication history for this paper can be accessed here:

http://www.biomedcentral.com/1472-6882/13/82/prepub
